# Integron-Mediated Antibiotic Resistance in* Acinetobacter baumannii* Isolated from Intensive Care Unit Patients, Babol, North of Iran

**DOI:** 10.1155/2017/7157923

**Published:** 2017-07-19

**Authors:** Mitra Deylam Salehi, Elaheh Ferdosi-Shahandashti, Yosef Yahyapour, Soraya Khafri, Abazar Pournajaf, Ramazan Rajabnia

**Affiliations:** ^1^Department of Microbiology, Faculty of Medicine, Babol University of Medical Sciences, Babol, Iran; ^2^Department of Medical Biotechnology, Faculty of Advanced Technologies in Medicine (SATiM), Tehran University of Medical Sciences, Tehran, Iran; ^3^Infectious Diseases and Tropical Medicine Research Center, Babol University of Medical Sciences, Babol, Iran; ^4^Department of Biostatistics and Epidemiology, Faculty of Medicine, Babol University of Medical Sciences, Babol, Iran; ^5^Department of Microbiology, Faculty of Medicine, Iran University of Medical Sciences, Tehran, Iran

## Abstract

**Background:**

We investigated the integron types and their relation with antibiotic resistance among* A. baumannii* isolates collected from intensive care unit patients, Babol, north of Iran.

**Methods:**

In this cross-sectional study, a total of 73 bronchoalveolar lavage samples were obtained from patients in ICU. Susceptibility testing was performed by disk diffusion method. Types of integrons were identified by an integrase gene PCR.

**Results:**

In total, 47.9%* A. baumannii* isolates were recovered from the BAL samples. All isolates were resistant to ceftazidime. 91.4% and 58.3% of isolates were MDR and XDR, respectively. The rate of colistin resistance with the *E*-test was 5.7%. Molecular analysis of class I, II, and III integrons showed that 25.7%, 88.6%, and 28.6% of the isolates carried the* intI*,* intII*, and* intIII* genes, respectively.

**Discussion:**

Our results show that different classes of integrons are commonly spread among* A. baumannii* strains and these genomic segments can play an important role in the acquisition of MDR and XDR phenotypes. So monitoring drug resistance in* A. baumannii* isolates with the use of* int* gene PCR is very important to plan specific infection control measures to prevent the spread of MDR-AB and XDR-AB in Iran's hospitals.

## 1. Introduction

Multidrug-resistant* Acinetobacter baumannii* (MDR-AB) is an important opportunistic pathogen responsible for severe hospital-acquired infections (HAIs), particularly patients admitted to the intensive care unit (ICU) [1]. MDR-AB infections especially pneumonia and bacteremia show a high mortality rate (30% to 75%) and require prolonged hospital stays in intensive care units (ICUs) [2]. In the ICU, the mortality rate associated with* A. baumannii* is 54%. The majority of infections are originated from epidemic outbreaks. Appearance of extensively drug-resistant* A. baumannii* (XDR-AB) limits the therapeutic options and causes a serious concern to HAI control. For a period of 5 years (2005–2010), the frequency of XDR-AB in clinical isolates was increased from 15% to more than 41%. Control of MDR-AB and XDR-AB infections is an important issue for clinical microbiologists and physicians. Treatment of* A. baumannii* has become difficult because many isolates are now resistant to a wide range of antimicrobial agents [3, 4].

An important factor that influences the development of multiresistance phenotype is the acquisition of mobile genetic elements (MGEs). Antibiotic-resistant elements in* Acinetobacter* strains are commonly carried on MGEs, such as the R plasmids, transposons (TEs), integrons* (Int)*, and genomic islands (GEIs) [5]. Integrons are conserved sequences (3′-CS and 5′-CS) of DNA that are able to acquire gene cassettes, which can carry antibiotic resistance genes, by site-specific recombination [6]. These genetic segments are considered by the presence of an* intI* gene (integrase), a recombination site (attI), and a promoter (P_C_) [7]. The most common types of integrons are the transportable class I (Tn402 derivatives) integron, followed by class II and class III integrons, respectively. Class I integrons harbor numerous antimicrobial resistance gene cassettes encoding broad-spectrum *β*-lactamase,* dfr* (dihydroflavonol-4-reductase/trimethoprim), qacEΔ1 (disinfectants and tetravalent ammonium compounds),* sul1* (sulfonamide), and aminoglycoside-modifying enzymes (AMEs). Integrons of class II are located in Tn7 and 3′-CS which contains five* tns* genes and are responsible for the mobility of TE. Integrons of class III located in transposons have also been described, but the 3′-CS is still not well defined [8].

MDR phenotype occurs in* A. baumannii* when different integrin-borne antibiotic resistance elements coexist, giving rise to MDR gene cassettes [9, 10]. Due to the high prevalence of this infection, as well as various profiles of drug resistance in different geographical areas, a study on the prevalence and antibiotic resistance pattern in different part of the world is essential. These data would provide useful information on the spread of resistance elements and the possibility of choosing appropriate treatment strategies. So, the aim of this study was to determine the frequency of type I, II, and III integrons in* A. baumannii* isolates recovered from bronchoalveolar lavage (BAL) samples obtained from hospitalized patients in ICU ward, Babol, north of Iran [11].

## 2. Materials and Methods

### 2.1. Setting

The Ayatollah Rouhani hospital is a 372-bed hospital and is one of the most equipped teaching therapeutic centers affiliated to the Babol Medical University in north of Iran. This study was approved by the ethical committee of Babol University of Medical Sciences for Research Analysis. In this analytical cross-sectional study, based on previous studies and confidence interval 95% and using equation *n* = *z*^2^*P*(1 − *P*)/*d*^2^, a total of 73 nonrepetitive and nonduplicative BAL samples were collected from ICU patients from May to November 2015.

### 2.2. Inclusion Criteria

*Clinical findings of pneumonia* are as follows: new radiographic infiltrate and two of the three subsequent criteria: fever [body temperature ≥ 38.0°C] or hypothermia temperature < 35.0°C [rqb], purulent bronchial secretion, and WBCs > 10,000/mm^3^ or <3,000/mm^3^ [12].

### 2.3. Exclusion Criteria

Contraindications to bronchoscopy include the following: (1) Po2 < 75 mm Hg even with an inspired oxygen fraction of 100%; (2) acute cardiac arrhythmia; and (3) bronchospasm [12].

### 2.4. Chest Radiograph (Chest X-Ray)

A portable chest radiograph was obtained prior to, and on the same day as, bronchoscopic sampling. Infiltration of lung (chest X-ray) was included as a criterion for hospital admission. The radiograph was reviewed and interpreted by a staff radiologist and pulmonary disease specialist [12].

### 2.5. Procedure and Specimen Collection (BAL Collection)

Patients were commonly sedated with IV midazolam before bronchoscopic procedure. The fraction of inspired oxygen was increased to 100%, and positive end-expiratory pressure was limited to 8 cm H_2_O. The bronchoscope used was the Pentax FB15X (Pentax Instruments, Tokyo, Japan). Bronchoscopic BAL samples were collected by pulmonologist by wedging the tip of a fiber optic bronchoscope in the subsegmental bronchus of the most compromised lobe seen in chest X-ray or, in cases of diffuse radiologic presentation, in the posterior bronchus of the lower lobe. As little topical lidocaine as possible was used so as not to interfere with bacterial growth (never >20 mg per bronchus). Aspiration of secretions by the bronchoscope was avoided. This procedure was repeated in the contralateral lung, and samples were preserved at ambient temperatures or 4°C before being transported to the microbiology laboratory within 2 h of collection [12, 13].

### 2.6. Quantitative Cultures

Quantitative cultures were processed according to the standard laboratory protocol. Sample pairs collected were diluted in tryptic soy broth (Merck, Co., Germany) plated at final dilutions of 10^−3^ and 10^−4^ onto eosin methylene blue agar, chocolate agar and blood agar (Merck, Co., Germany) plates and then incubated at 35°C under 5% CO_2_. Gram staining was performed on 1 drop of undiluted BAL fluid. Final colony counts were determined after 48 h. Potential pathogens present at ≥1 × 10^4^ CFU/ml were considered clinically significant, and subsequent identification and antimicrobial susceptibility testing were performed. Isolates detected at ≥1 × 10^3^ and <1 × 10^4^ CFU/ml were presumptively identified, and antimicrobial susceptibility testing was not performed. The following were considered etiologic agents of pneumonia: (1) bacteria with >10^4^ CFU/mL in BAL quantitative culture (according to clinical judgment, >10^3^ CFU/mL was considered positive) and (2) demonstration by direct microscopic examination of BAL fluid [12, 13].

### 2.7. Bacterial Isolates

After transferring all samples to the Department of Microbiology in the medicine faculty,* A. baumannii* strains were recognized based on conventional biochemical and microbiological tests including Gram staining, oxidase and catalase test, motility, oxidation of glucose, hydrolysis of esculin, decarboxylation of lysine, hydrolysis of arginine, reduction of nitrate, citrate utilization, oxidative/fermentative glucose (O/F) test, and growth ability at 44°C. Negative result for oxidase test, no motility, nonfermentation, and growth in temperature of 42–44°C were considered as the elementary criteria for* A. baumannii* recognition [14]. In order to confirm the identity of strains, the presence of* gyrB* gene was assessed using PCR [15, 16]. The isolates were preserved in −80°C in brain-heart infusion broth (Merck, Co., Germany) containing glycerol 50% v/v until the molecular analysis.

### 2.8. Antimicrobial Susceptibility Testing

Antimicrobial susceptibility testing was performed on Mueller-Hinton agar (MHA) (Merck, Co., Germany) by agar disk diffusion (DD) method as recommended by the Clinical and Laboratory Standards Institute (CLSI document M100-S14) [17]. The tested antibiotics were as follows: amikacin (AK; 30 *μ*g), ciprofloxacin (CP; 5 *μ*g), ceftazidime (CAZ; 30 *μ*g), gentamicin (GM; 10 *μ*g), imipenem (IMP; 10 *μ*g), meropenem (MER; 10 *μ*g) and piperacillin/tazobactam (PTZ; 100/10 *μ*g) (MAST diagnostics, Merseyside, UK).* A. baumannii* ATCC 17978 was used as a positive quality control (PQC) and* P. aeruginosa* ATCC 25853 and* E. coli* ATCC 25922 were used as a negative quality control (NQC) in this study.

### 2.9. Minimum Inhibitory Concentration (MIC)

All isolates were tested for colistin (CS) susceptibility by *E*-test according to the manufacturer's guidelines (Liofilchem SRL, Italy). Suspension of each isolate in Mueller-Hinton broth, adjusted to the density of a 0.5 McFarland standard, was swabbed in three directions to ensure uniform growth onto Mueller-Hinton agar plates. An *E*-test CS strip (ranging from 0.016 to 256 mg/ml) with interpretative criteria (susceptible (S), ≤2 *μ*g/ml; resistant (R), ≥4 *μ*g/ml) was used for each plate when the agar surface was completely dry, and the plates were incubated at 35°C for 16–20 h. The minimum inhibitory concentration (MIC) value was considered at the point of complete inhibition of all growth, including hazes. The interpretive criteria used were those established in Clinical and Laboratory Standards Institute standard [14].

### 2.10. PCR Amplification and Sequencing

The PCR technique were performed by the DNA amplification device master cycler gradient (Eppendorf Co., Hamburg, Germany) for detection of* intI-I*,* intI-II,* and* intI-III* genes. Whole-cell (genomic) DNA was extracted from each strain using a high pure PCR template preparation kit (Roche Co. in Germany). The integron encoding gene classes and PCR programs are listed in Table 1. PCR was performed in a whole volume of 25 *μ*l. PCR mixture contained 2 *μ*l of 10x PCR buffer, 1.5 DNA template, 0.8 *μ*l MgCl_2_, 0.6 *μ*l dNTPs, 1.5 *μ*l of each primer, 0.7 IU of Taq DNA polymerase (Ampliqon Co., Denmark), and 14.9 *μ*l of sterile distilled water. Amplification products were electrophoresed on 1.5% agarose gels at 5 V/cm. Gels were stained with ethidium bromide (EtBr) (0.5 *μ*g/ml) and visualized on a UV trans-illuminator (Vilber Lourmat, Cedex, France). The amplicon sizes were determined by comparison with a DNA size marker (100-bp DNA ladder, Fermentas). Direct sequencing of the PCR products was performed in both directions using an ABI3730XL DNA analyzer (Applied Biosystems, Forster, USA). Nucleotide sequence data were analyzed at the National Center for Biotechnology Information (NCBI), available at the website (http://blast.ncbi.nlm.nih.gov/Blast.cgi).

### 2.11. Statistical Analysis

SPSS version 23 (SPSS, Inc., Chicago, IL, USA) was employed for statistical analysis. Descriptive statistics and Pearson's chi‐square tests were used to evaluate the correlation between various classes of integrons and antimicrobial resistance. Statistical significance was defined as *P* value less than 0.05.

## 3. Results

Mean of patients ages was 54.5 years (range, 17 to 92 years). 32 (43.8%) of them were female and 41 (56.2%) were male. The mean APACHE II (acute physiology and chronic health evaluation II) index was 16.2 at ICU admission (range, 7 to 31). Mean duration of hospitalization until BAL performance was 14.3 days (range, 4 to 48 days). The main reasons for admission to ICU were as follows: neurologic emergencies in 23 patients (31.5%); respiratory failure in 19 (26.0%); cardiocirculatory emergencies in 14 (19.2%); other infections in 3 (4.1%); hematologic disorders in 3 (4.1%); trauma in 3 (4.1%); septic shock in 2 (2.7%); renal failure in 2 (2.7%); diabetes in 2 (2.7%); malignancy in 1 (1.4%), and acute abdomen in 1 (1.4%).

Forty-nine of 73 BALs (67.1%) presented >10^4^ CFU/mL in BAL quantitative culture. The three BALs (4.8%) that presented between 10^3^ and 10^4^ CFU/mL were also considered positive. Total positivity was 52 of 73 (71.2%); 49 of these 52 positive (67.1%) had >10^4^ CFU/mL. There were no cases of bacterial growth with <10^3^ CFU/mL. Twenty-one cases (28.7%) did not present bacterial growth (Table 2).

**Figure 1:**
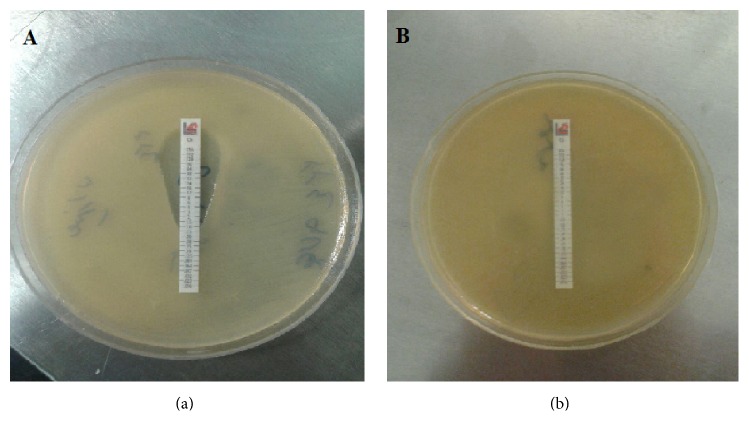
*E*-test MIC value of colistin. (a) Colistin-susceptible* A. baumannii* (interpretative criteria; susceptible (S), ≤2 *μ*g/ml). (b) Colistin-resistant* A. baumannii* (interpretative criteria; resistant (R), ≥4 *μ*g/ml). Colistin sulfate (CS), ranging from 0.016 to 256 mg/ml produced by Liofilchem SRL, Roseto degli Abruzzi, Italy.

*A. baumannii* isolates (*n* = 35; 47.9%) were recovered from the BAL samples (*n* = 73). The highest resistance rate was related to CAZ (100%) (Table 3). In the current study, MDR-AB is defined as resistance to more than three classes of antibiotics. XDR-AB is regarded as the isolate that is resistant to 3 classes of antibiotics defined above (MDR) as well as to carbapenems. Finally, pan-drug-resistant* A. baumannii* (PDR-AB) is the XDR isolate that is also resistant to tigecycline and polymyxins. According to the results of the antimicrobial susceptibility test, 91.4% and 58.3% of the isolates were MDR and XDR. The rates of CS resistance with the *E*-test method in all* A. baumannii* studied strains were determined 5.7% (*n*; 2 of 35) (Figure 1).

Molecular detection of class I, II, and III integrons was performed by amplification of* intI*,* intII*, and* intIII* genes in all* A. baumannii* strains using PCR method. The results revealed that 94.3% (*n*; 33/35) of the isolates carried the* int* gene. Molecular analysis of class I, II, and III integrons showed that 25.7% (*n*; 9), 88.6% (*n*; 31), and 28.6% (*n*; 10) of isolates carried the* intI*,* intII,* and* intIII* genes, respectively. The coexistence of* intI/intII* and* intI/intII/intIII* in isolates was in order 22.9% (*n*; 8) and 8.6% (*n*; 3). Two (5.7%) of all isolates were* int*-negative. The correlation of class I, II, and III integrons and antibiotic resistance profile are shown in Table 4. Nucleotide sequence data reported in the current study has been deposited in the PubMed/NCBI/GenBank nucleotide sequence databank under the accession numbers KX122025.1, KX122026.1, KX122027.1, KX122028.1, and KX122029.1.

## 4. Discussion

*A. baumannii* is an important opportunistic bacterium causing severe HAIs with high mortality rates due to its wide drug resistance. Integrons are vital in enabling the* A. baumannii* genome to capture and accumulate many antimicrobial resistance genes. Because of the MDR phenotype and the trend to spread in the hospital environment,* A. baumannii* has a special clinical significance requiring epidemiological monitoring as a measure to control the spread of HAIs [18].

In line with Gomes et al. [12], Zaccard et al. [13], and Nomanpour et al. [19] studies, considering 10^4^ CFU/mL as the cutoff and including the cases with bacterial growth between 10^3^ and 10^4^ CFU/mL, 28 of all 52 positive BALs (53.8%) presented more than one isolate, which means polymicrobial infections. The most frequently encountered pathogens were associated with two different strains of* A. baumannii* (48%) and between* P. aeruginosa* (5.5%) and MRSA (5.5%), with three cases of each association.

Our findings showed that all these strains were resistant to CAZ. DD method results showed 91.4% and 58.3% of the isolates were MDR and XDR, respectively. These results are in agreement with the Ghajavand et al. study [20]. In a parallel study piloted in Taipei, Taiwan, 25 XDR-AB were found during two years of study [21]. This contrast may be due to the geographical distance and/or the different levels of hygiene. The majority of the strains were sensitive to CS, regarded as the last resort against MDR-AB, probably due to the fact that CS has not been broadly used in Iran; thus, the corresponding selection pressure has not been established on this bacterial species. *E*-test MIC value showed that 5.7% (*n*; 2) of isolates were resistant to CS. This data are similar to Vakili et al. [14]. All CS-resistant isolates were positive for class II integron. No significant association was observed between CS-resistant isolates and class II integron.

Integrase-encoding genes were found in 33 (94.3%) of the 35 isolates. Our data showed that the prevalence of class I, II, and III integrons was 25.7%, 88.6%, and 28.6%, respectively. In the strains that had all the three classes of integrons, resistance to all the antibiotics tested was observed. Two samples (5.71%) did not contain any integrons but were still resistant to CP and CAZ, which indicates that resistance genes could also be transmitted by other elements such as plasmids, TEs, and bacteriophages. In the study carried by Ramírez et al., the frequency of class I and II integrons was 42% and 68%, respectively [22]. Also, Kamalbeik et al. showed that 7.5% and 67.5% of the strains contained class I and II integrons, respectively [23]. In the studies carried out by Ramírez et al. [24] and Martins et al. [25], the prevalence of class II Integron was 23% and 41.7%, respectively. In the research conducted by Taherikalani et al. (Tehran at 2012) [26] and Moammadi et al. (west of Iran at 2012) [27] the frequency of class I, II, and III integrons was 85%, 14%, and 0%, and 97%, 31%, and 0%, respectively. These findings are in contrast with our results and may be related to the difference in source of samples, level of hygiene, and geographical areas. So, it might be due to stiff search-and-destroy and surveillance policies, as well as control in antibiotic prescription.

Integron-positive* A. baumannii* isolates showed higher antibiotic resistance rate compared to integron-negative strains. All the isolates with class II integron had a significant relationship with resistance to all the tested antibiotics which could be due to the gene cassettes that encoded resistance to these antibiotics. According to our results, there was a significant correlation between the presence of class I, II, and III integrons and resistance to CAZ. Lin et al. [28] and Mirnejad et al. [29] observed a significant correlation between the presence of integrons and resistance to CP, ofloxacin, cefepime, CAZ, aztreonam, AN, and norfloxacin. Koeleman et al. indicated a significant relationship between the presence of integrons and resistance to AN, CP, and CAZ [30]. The results of Gaur et al. confirmed the results of our study and showed correlation between the presence of integrons and resistance to AN, cefepime, and CP [31]. In cases where significant relationships between the integrons and antimicrobials resistance were not detected, resistance could be attained by resistance elements that reside on MGEs, such as plasmids, TEs, and phages, indicating substantial HGT from other bacteria [32].

## 5. Conclusion

In general, our results showed that different classes of integrons are commonly spread among* A. baumannii* strains and these genomic segments can play an important role in the acquisition of MDR and XDR* A. baumannii*. In this study, regardless of the presence or lack of resistance genes, strong association was observed between the presence of class II integrons and a decreased sensitivity to the many classes of antibiotics which could be challenging, since these structures can move among strains and consequently become resistant to new antimicrobials. So, monitoring drug resistance in* A. baumannii* isolates with the use of* int* gene PCR is very important to plan specific infection control measures to prevent the spread of MDR-AB and XDR-AB in Iran's hospitals.

## Figures and Tables

**Table 1:** Primer sequences, PCR product sizes, and PCR programs for replication of class I, II, and III integrons.

Genes	Primer sequence (5′ → 3′)	PCR product (bp)	PCR program	Reference
*IntI-I*	F-TCTCGGGTAACATCAAGG R-AGGAGATCCGAAGACCTC	243	5 min at 94°C; 35 cycles (1 min at 94°C, 1 min at 53°C, and 30 sec at 72°C); 5 min at 72	[9]

*IntI-II*	F-TTATTGCTGGGATTAGGC R-ACGGCTACCCTCTGTTATC	233	5 min at 94°C; 30 cycles (1 min at 94°C, 1 min at 50°C, and 1 min at 72°C); 10 min at 72°	[10]

*IntI-III*	F-AGTGGGTGGCGAATGAGTG R-TGTTCTTGTATCGGCAGGTG	600	5 min at 94°C; 30 cycles (1 min at 94°C, 1 min at 50°C, and 1 min at 72°C); 10 min at 72°C	[10]

**Table 2:** Bacterial etiologic agents.

Agents	All BALs		
	Mono (%)	Poly (%)	Total (%)
*A. baumannii*	30 (41.1%)	5 (6.8%)	35 (48%)
*Pseudomonas aeruginosa*	3 (4.1%)	1 (1.9%)	4 (5.5%)
*Staphylococcus aureus*	2 (2.7%)	2 (2.7%)	4 (5.5%)
*Klebsiella pneumoniae*	1 (1.4%)	0 (0.0%)	1 (1.4%)
*Escherichia coli*	0 (0.0%)	1 (1.4%)	1 (1.4%)
*Enterobacter cloacae*	0 (0.0%)	1 (1.4%)	1 (1.4%)
*Serratia marcescens*	0 (0.0%)	2 (2.7%)	2 (2.7%)
*Stenotrophomonas maltophilia*	1 (1.4%)	0 (0.0%)	1 (1.4%)
MRCoNS	1 (1.4%)	0 (0.0%)	1 (1.4%)
*Streptococcus pneumoniae*	2 (2.7%)	0 (0.0%)	2 (2.7%)

Mono = unique isolated agent; Poly = polymicrobial infection; MRCoNS = methicillin resistant coagulase-negative Staphylococcus.

**Table 3:** Antibiotics resistance patterns among *Acinetobacter* isolates.

Antibiotics	R (%)	I (%)	S (%)
Amikacin (AN)	32 (91.4)	0 (0.0)	3 (8.6)
Gentamicin (GM)	30 (85.7)	1 (2.9)	4 (11.4)
Ciprofloxacin (CP)	33 (94.3)	0 (0.0)	2 (5.7)
Ceftazidime (CAZ)	35 (100)	0 (0.0)	0 (0.0)
Imipenem (IPM)	33 (94.3)	2 (5.7)	0 (0.0)
Meropenem (MEN)	33 (94.3)	1 (2.9)	1 (2.9)
Piperacillin-tazobactam (PIP-TZ)	32 (91.4)	1 (2.9)	2 (5.7)

I = intermediate; R = resistance; S = susceptible.

**Table 4:** The correlation of class I, II, and III integrons and antibiotic resistance profile.

Antibiotics	Positive for integron class I (*n*; 9)	Negative for integron class I (*n*; 26)	*P* value	OR (95 CI)	Positive for integron class II (*n*; 31)	Negative for integron class II (*n*; 4) 8	*P* value	OR (95 CI)	Positive for integron class III (*n*; 10)	Negative for integron class III (*n*; 25)	*P* value	OR (95 CI)
AN												
R	9 (25.7%)	23 (65.7)	0.287	1.666 (0.138–90.16)	30 (85.7)	2 (5.7)	0.002	30 (0.915–182)	10 (28.6)	22 (62.9)	0.252	1.913 (0.159–102)
I	0 (0)	0 (0)			0 (0)	0 (0)			0 (0)	0 (0)		
S	0 (0)	3 (8.6)			1 (2.9)	2 (5.7)			0 (0)	3 (8.6)		

GM												
R	7 (20)	23 (65.7)	0.430	2.190 (0.303–15.851)	28 (80)	2 (5.7)	0.030	9.33 (0.457–159.29)	10 (28.6)	20 (57.2)	0.127	3.142 (0.307–157.47)
I	1 (2.9)	0 (0)			1 (2.9)	0 (0)			0 (0)	1 (2.9)		
S	1 (2.9)	3 (8.6)			2 (5.7)	2 (5.7)			0 (0)	4 (11.4)		

CP												
R	8 (22.6)	25 (71.4)	0.418	3.125 (0.175–55.886)	31 (88.6)	4 (11.4)	0.000	31 (1.576–1675.56)	9 (25.7)	24 (68.6)	0.357	1.375 (0.963–78.648)
I	0 (0)	0 (0)			0 (0)	0 (0)			0 (0)	0 (0)		
S	1 (2.9)	1 (2.9)			0 (0)	0 (0)			1 (2.9)	1 (2.9)		

CAZ												
R	9 (25.7)	26 (74.3)	0.004	—	31 (88.6)	4 (11.4)	0.000	—	10 (28.6)	25 (71.4)	0.011	—
I	0 (0)	0 (0)			0 (0)	0 (0)			0 (0)	0 (0)		
S	0 (0)	0 (0)			0 (0)	0 (0)			0 (0)	0 (0)		

IPM												
R	8 (22.9)	25 (71.4)	0.418	3.125 (0.175–55.886)	31 (88.6)	2 (5.7)	0.000	32 (2.484–15570.62)	9 (25.7)	24 (68.5)	0.490	2.6667 (0.150–47.302)
I	1 (2.9)	1 (2.9)			0 (0)	2 (5.7)			1 (2.9)	1 (2.9)		
S	0 (0)	0 (0)			0 (0)	0 (0)			0 (0)	0 (0)		

MER												
R	9 (25.7)	24 (68.6)	0.392	1.2 (0.836–69.206)	31 (88.6)	2 (5.7)	0.000	32 (2.484–15570.62)	10 (28.6)	23 (65.7)	0.357	1.375 (0.963–78.648)
I	0 (0)	1 (2.9)			0 (0)	1 (2.9)			0 (0)	1 (2.9)		
S	0 (0)	1 (2.9)			0 (0)	1 (2.9)			0 (0)	1 (2.9)		

PIP-TZ												
R	8 (22.9)	24 (68.6)	0.757	1.50 (0.119–18.836)	31 (88.6)	1 (2.9)	0.000	32 (2.484–15570.62)	9 (25.7)	23 (65.7)	0.849	1.278 (0.103–15.899)
I	0 (0)	1 (2.9)			0 (0)	2 (5.7)			0 (0)	1 (2.9)		
S	1 (2.9)	1 (2.9)			0 (0)	0 (0)			1 (2.9)	1 (2.9)		

AN: amikacin, GM: gentamicin, CP: ciprofloxacin, CAZ: ceftazidime, IPM: imipenem, MER: meropenem, PIP-TZ: piperacillin-tazobactam, R: resistance, I: intermediate, S: sensitive, and OR: odds ratio.
